# The Recent Development in the Diagnosis of *Mycobacterium tuberculosis*

**DOI:** 10.1002/smmd.70007

**Published:** 2025-04-25

**Authors:** Nimet Temur, Esma Eryilmaz‐ Eren, Ilhami Celik, Ilknur E. Yıldız, Mustafa Nisari, Ilay S. Unal, Cagla Celik, Nilay Ildiz, Ismail Ocsoy

**Affiliations:** ^1^ Faculty of Pharmacy Department of Analytical Chemistry Erciyes University Kayseri Türkiye; ^2^ Kayseri City Training and Research Hospital University of Health Science Kayseri Türkiye; ^3^ Faculty of Medicine Department of Infectious Diseases and Clinical Microbiology Recep Tayyip Erdogan University Rize Türkiye; ^4^ Faculty of Dentistry Department of Medical Biochemistry University of Nuh Naci Yazgan Kayseri Türkiye; ^5^ Faculty of Pharmacy Department of Pharmaceutical Technology Zonguldak Bulent Ecevit University Zonguldak Türkiye; ^6^ Faculty of Pharmacy Department of Analytical Chemistry Harran University Sanliurfa Türkiye; ^7^ Medical Imaging Department Vocational School of Health Services Bandırma Onyedi Eylul University Bandirma Türkiye

**Keywords:** diagnosis, *Mycobacterium tuberculosis*, rapid test

## Abstract

*Mycobacterium tuberculosis* (MTB) remains a global health issue and continues to rank among the leading causes of death from infectious diseases worldwide. Its persistence is primarily attributed to the microorganism's challenging and time‐consuming diagnosis and treatment, which drives the need for new diagnostic tests. The development of rapid, highly sensitive point‐of‐care (POC) tests is crucial, as these tests address the limitations of traditional methods, which are lengthy and exhibit low sensitivity. Early and rapid diagnostic tests ensure timely diagnoses and treatments for individuals while playing a pivotal role in preventing the spread of MTB and curbing societal transmission. These diagnostic tests significantly impact TB diagnosis and treatment, potentially breaking the chain of transmission and presenting a promising step toward combating the infection. Rapid and accurate diagnostic tests for MTB detection continue to attract significant attention in the literature and show promise for widespread application. However, they face challenges such as limited accessibility and usability, particularly in underdeveloped countries. The implementation of rapid tests requires careful consideration of time and resource efficiency compared with traditional tests. This study reviews the diagnostic tests developed for MTB detection, tracing their evolution from the past to the present.

## Graphical abstract

*Mycobacterium tuberculosis* (MTB) remains a major global health challenge due to slow, low‐sensitivity traditional diagnostics. Rapid, highly sensitive point‐of‐care tests are critical for timely diagnosis and treatment, reducing transmission. This study reviews the evolution of MTB diagnostic tests, emphasizing advancements and addressing accessibility and efficiency challenges.

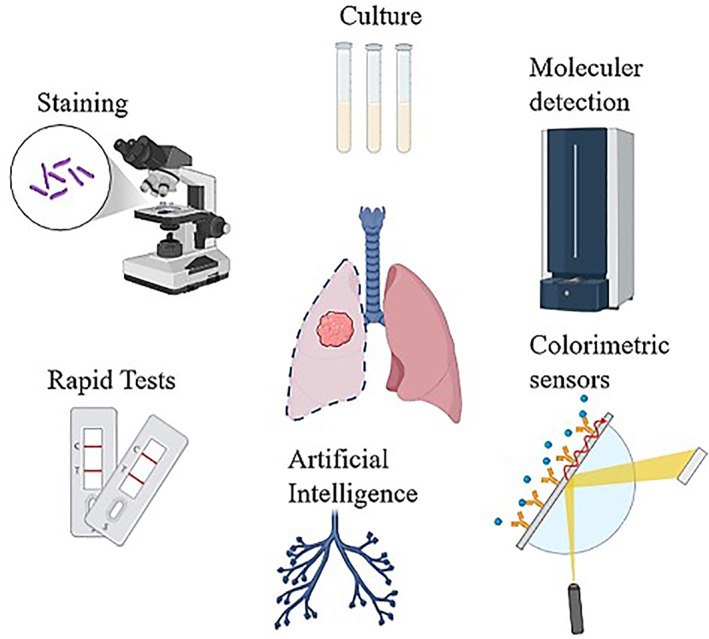

**Summary**

- Explanation of conventional and molecular methods for the detection of Mycobacterium tuberculosis (MTB) from past to present.
- Recent developments in nanomaterial‐based, rapid, and artificial intelligence‐supported diagnostic tests for the diagnosis of MTB are discussed.

## 1 Introduction

*Tuberculosis* (TB) is among the infectious diseases with the highest mortality and morbidity rates globally, causing numerous new cases and approximately 1.5 million deaths annually [1]. Furthermore, the increasing incidence of HIV and AIDS has contributed to this rise in TB cases [2]. Globally, TB is the second leading cause of death among infectious diseases, surpassing the death rate of AIDS patients [1]. In particular, societal transmission of TB is widespread in underdeveloped and developing countries due to limited access to diagnostic and treatment tests [1, 2].

TB is caused by *Mycobacterium tuberculosis* (MTB) and primarily affects the lungs but is a multisystemic disease. MTB can infect the brain, kidneys, and other parts of the body besides the lungs [3]. The MTB complex includes seven different bacterial species: *Mycobacterium tuberculosis* (*M*. *tuberculosis*, MTB), *Mycobacterium bovis* (*M*. *bovis*), *Mycobacterium africanum* (*M*. *africanum*), *Mycobacterium microti* (*M*. *microti*), *Mycobacterium canettii* (*M*. *canettii*), *Mycobacterium caprae* (*M*. *caprae*), and *Mycobacterium pinnipedii* (*M*. *pinnipedii*) [4]. MTB is an aerobic, non‐spore‐forming, non‐motile *bacillus* and humans are its only host. The bacterial cell wall is composed of mycolic acid, arabinogalactan, and peptidoglycan and contains high levels of lipids [5]. This high lipid content is significant because it makes the bacteria resistant to antibiotics due to the thick and durable cell wall, posing a major challenge in TB treatment [6]. Additionally, it facilitates acid‐fast staining and increases virulence. Identifying the components of MTB's cell wall remains a key area of research for rapid diagnostic tests.

MTB is easily transmitted through aerosols, infecting healthy individuals via respiration. The primary mode of TB transmission is airborne, particularly when an infected individual coughs or sneezes [7]. Prevention strategies, such as proper ventilation and isolating infected individuals in negative‐pressure single‐occupancy rooms, are essential for controlling the disease's spread. Individuals in close contact with active TB patients, such as household members or those sharing enclosed spaces, are at a high risk of infection. Understanding TB transmission pathways and implementing prevention strategies are essential for early and rapid diagnosis. Rapid and accurate detection of bacilli is crucial for breaking the chain of transmission and preventing disease progression. Rapid diagnostic tests are considered as key not only for early diagnosis and treatment but also for halting the infection spread and effectively combating the infection [8, 9, 10].

Currently, MTB diagnostic tools remain limited in underdeveloped and developing countries, where traditional methods such as microscopic staining and culture are still widely used. Due to its high mycolic acid content, MTB is acid‐resistant and can be detected microscopically using acid‐fast staining methods. Culture, another traditional test, is still considered the gold standard in diagnosis. However, these methods have low sensitivity, often yielding false‐negative results and allowing the infection to spread. Traditional methods are also labor‐intensive and time‐consuming. Moreover, they cannot differentiate latent TB from active TB, which is significant because latent TB can progress to active TB. This progression is particularly concerning in immunosuppressed patients, such as those with HIV, where early diagnosis and prophylaxis are critical [11, 12, 13, 14].

**FIGURE 1:**
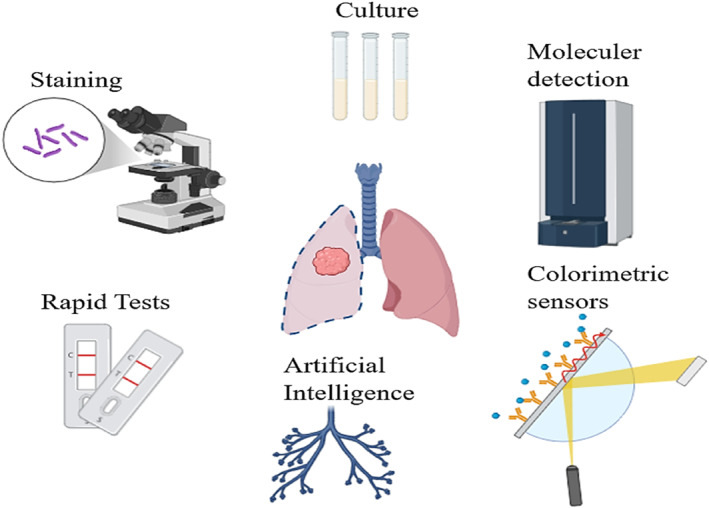
Schematic illustration of developed methods for the diagnosis of TB (created with BioRender.com).

Molecular biological and immunological diagnostic methods are also employed but pose challenges due to their expensive equipment requirements, lengthy procedures, and laborious nature, creating obstacles in diagnosis and treatment [15]. All these diagnostic methods for TB have disadvantages in terms of feasibility and cost. Rapid and accurate detection of bacilli is critical for breaking the chain of transmission. Among these tests, rapid diagnostic tests stand out for their promise in enabling individuals to receive early diagnosis and treatment, preventing societal transmission, and offering a more hopeful outlook in combating TB. It is anticipated that research in this area will increase in the future. The methods that have been developed for the diagnosis of TB are summarized in Figure 1.

## 2 Conventional Diagnostic Methods

Diagnosis of tuberculosis is a complex process, often hindered by its non‐specific symptoms. Beyond history and physical examination, basic tests are usually necessary. For instance, lung imaging is crucial for diagnosing pulmonary tuberculosis [16]. While a clinician can make a “clinical diagnosis” based on history, physical examination, and clinical findings, bacteriological diagnosis is considered the gold standard for a “definitive diagnosis” method. This involves demonstrating bacilli in culture from sputum or samples taken from the affected organ, providing a definitive diagnosis of tuberculosis. Acid‐fast staining and Nucleic Acid Amplification Tests (NAATs) also support the diagnosis [11, 17]. Recent estimates show that around 1.7 billion people are latently infected with MTB at the same time, traditional diagnostic methods, such as chest X‐ray and TB skin tests, are not sufficiently sensitive and specific to reliably diagnose latent forms of TB, especially against the background of other diseases or pathological conditions. The risk of progression of latent TB infection to the active form is estimated at 10%. In patients with latent tuberculosis infection (LTBI), in potentially immunosuppressive conditions, prophylactic treatment is planned to prevent progression to active disease. In LTBI, bacteriological diagnosis is impossible, so indirect diagnostic tests are used [18].

### 2.1 Culture

**FIGURE 2:**
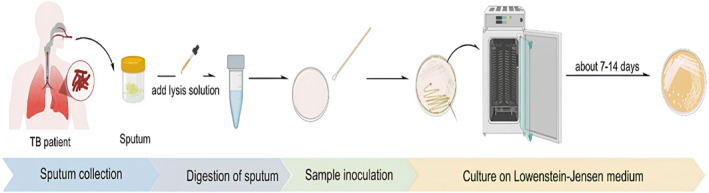
Schematic illustration of the gold standard culture for the diagnosis of TB. Re‐used with permission [24]. Copyright 2023, American Chemical Society.

There are three types of traditional culture media: egg‐based (Lowenstein‐Jensen), agar‐based (Middlebrook 7H10 or 7H11), and liquid (Middlebrook 7H12 and other commercially available broths). After culture growth, further investigation for species identification and drug susceptibility testing is recommended. The advent of rapid culture techniques, a crucial development in the field, has significantly reduced the time required for culture growth. These techniques, now possible with liquid media and fluorescence‐based systems, have brought a new efficiency level to the process. The most common is the commercially available Bactec Mycobacteria Growth Indicator Tube (MGIT) (Becton Dickinson, Sparks, Massachusetts) system, which includes first‐line drug susceptibility testing [19, 20]. The Microscopic Observation Drug Susceptibility (MODS) test is also used to confirm drug susceptibility for second‐line drugs in cases of multidrug‐resistant tuberculosis (MDR TB) [21]. The rapid diagnostic tests group also includes tests based on the principle of color change in vitro growth. At this point, firstly, the nitrate reductase assay (NRA) with the calorimetric method, the modified NRA (NRAp) using para‐nitrobenzoic acid (PNB), and the resazurin tube test (RETAp) using PNB were studied. The results were reached in an average of 10 days. However, the sensitivity of the tests was found to be below 80% [22, 23]. Current research is focused on this issue. Figure 2 illustrates the culture method, which is considered the gold standard for diagnosing TB [24].

### 2.2 Staining

The quickest and the least expensive means for diagnosing TB is to detect acid‐fast bacilli (AFB) in microscopic examination of stained sputum smears. Acid‐fast staining is based on the principle of staining the lipid‐rich cell walls of mycobacteria with the decolorizing agent acid alcohol. It is prepared using carbofuchsin, acid alcohol, and methylene blue. Approximately 10,000 bacilli per mL are required to detect bacteria in an AFB smear. Since non‐mycobacterial microorganisms such as non‐tuberculous mycobacteria and Nocardia are also stained with AFB, its sensitivity is 45%–80%, and its positive predictive value is approximately 50%–80%. Sensitivity, which can reach up to 90%, varies according to sample type, infecting mycobacteria species, staining type, smear thickness, sample concentration, and number of samples [25, 26].

## 3 Molecular Diagnostic Methods

NAATs, with their ability to diagnose by detecting MTB DNA swiftly and accurately in clinical samples, provide a sense of reassurance regarding the speed of TB diagnosis. Among the various NAATs recommended by the World Health Organization (WHO), the most commonly utilized for TB diagnosis and detecting rifampicin resistance is GeneXpert MTB/RIF, with its upgraded version being GeneXpert Ultra [27, 28].

NAATs are used to rapidly diagnose organisms belonging to the MTB complex in patients with suspected TB, usually within 24–48 h. NAATs have been investigated for a long time. However, they are generally less sensitive than culture because the number of MTB in clinical specimens is relatively low, dead organisms may test positive, and DNA from resistant mycobacteria is challenging to extract effectively [29].

Tests that include line probe assay (LPA) and loop‐mediated isothermal amplification (LAMP) techniques allow the detection of MTB and the determination of resistance to rifampicin and isoniazid [30, 31]. It can be used in combination with acid‐fast bacilli (AFB) positivity to diagnose active TB. However, it is not used for treatment monitoring. Additionally, a negative NAA result is not sufficient to exclude active TB or drug resistance [31]. The GeneXpert MTB/RIF was introduced in 2010 and can furnish results in about 2 h, and is effective in pulmonary TB detection with a reported sensitivity between 85% and 90% with a specificity of almost 98%. Diagnostic purposes are aided particularly where there is a high prevalence of TB, and there is a need to find resistance to rifampicin (rifampin resistance‐determining region of the rpoB gene), the most critical indicator of MDR‐TB [28]. WHO has approved the Xpert MTB/RIF test for diagnosing both pulmonary and extrapulmonary TB and the MTBDR‐plus line‐probe test for diagnosing pulmonary TB. MTBDR‐plus is a molecular line‐probe assay that can detect rifampin and isoniazid resistance mutations (rpoB gene for rifampin resistance; katG and inhA genes for isoniazid resistance). The test's sensitivity is equal to GeneXpert MTB/RIF devices; the test detects 85%–90% +, and the specificity is approximately 98%. It was easy to perform the test in less than 1 h, and it was suitable and especially beneficial in outdoor settings with no stable source of electricity [32, 33].

Several molecular‐based commercial tests have been developed to detect mycobacterial nucleic acids in sputum or culture samples. For example, the COBAS TaqMan MTB Assay (Roche Diagnostics, Switzerland) detects MTBC DNA in respiratory samples. The AMPLIFIED MTD test (Gen‐Probe, San Diego, CA, USA) is a DNA probe technology for detecting MTBC in sputum by detecting MTBC rRNA. The sensitivity of these tests is over 90%, even in smear‐negative tuberculosis [34, 35]. LAMP test has been licensed as a molecular technique and made available as an easier‐to‐appreciate NAAT method than smear microscopy. It performs in less than 2 h, its sensitivity is approximately 80%, and its specificity is 98%–99%. TB‐LAMP is especially beneficial in low‐resource areas where large apparatus is out of reach [36, 37, 38].

## 4 Immunological Tests

The immune response to these antigens can show that a person has latent TB infection using immunological methods such as Interferon‐gamma release assays (IGRAs). Such tests, with their greater specificity than the traditional TST, instill confidence in their accuracy, albeit with limited efficacy, even among the BCG‐vaccinated population; these tests are used in active and latent TB diagnosis but not to differentiate between active and latent TB.

### 4.1 Tuberculin Skin Test

The first and oldest of the indirect diagnostic tests is the tuberculin skin test (TST). It detects cell‐mediated immunity to MTB by a delayed‐type hypersensitivity reaction employing a protein precipitate of heat‐inactivated tubercle bacilli (tuberculin as purified protein derivative [PPD]). TST is administered into the volar surface of the forearm by intradermal injection of 0.1 mL of PPD (5 TU) (Mantoux method). After 48–72 h, the transverse diameter of the palpable induration is measured to interpret the test [39, 40]. TST interpretation is classified according to risk. A skin reaction of 5 mm or greater is considered positive for close contact with cases of tuberculosis, persons with HIV infection, immunosuppressed individuals, persons with evidence of current or past TB, and those receiving TNF‐blocking agents. A reaction of ≥ 10 mm is considered positive for other people at increased risk for LTBI (e.g., persons born in countries with high incidence of TB and persons at risk for occupational exposure to TB) and persons with medical risk factors that increase the likelihood of progression from LTBI to TB. A reaction of 15 mm or greater is considered positive for all other people [41, 42].

False adverse reactions are the most critical limitations of TST, such as in infants and young children, in the early post‐infection period (< 6–8 weeks), in persons who have recently received live virus vaccines (measles, varicella, etc.), in immunocompromised patients or after recent viral (HIV, measles) and bacterial infections (typhoid, brucellosis) [43].

### 4.2 Interferon‐Gamma Release Assays

IGRA tests use IFN‐gamma secreted from T cells against *M*. *tuberculosis* antigens. However, they cannot detect active diseases. QuantiFERON‐TB Gold (QFT‐G) test, which belongs to the IGRAS group and is provided for diagnosing latent TB infection, has about 40% overall accuracy. Its sensitivity is almost 75 − 85%, and specificity is 98–99% [44, 45]. It cannot be applied for the diagnosis of active TB as no pathological differences of interest are embedded with the assay method; however, latent TB is diagnosed [31, 36].

Region of Difference‐1 (RD‐1) has been identified in the *M*. *tuberculosis* genome. This DNA region encodes two proteins: “Early Secretory Antigenic Target‐6” (ESAT‐6) and “Culture Filtrate Protein‐10” (CFP‐10). They are absent in *M*. *marinum*, *M*. *kansasii*, *M*. *szulgai*, and most non‐tuberculous mycobacteria except *M*. *flavescens* and the BCG vaccine strain [46, 47]. Their absence in BCG eliminates false positive results from the vaccine. QUANTIFERON‐TB gold in Tube and QUANTIFERON‐TB gold plus tests were developed using ESAT‐6 and CFP‐10, specific antigens for MTB [48].

T‐SPOT. TB is another self‐contained IGRA T‐SPOT. TB still possesses a slightly higher sensitivity of approximately 90% compared to QuantiFERON and a specificity of 98%. It is similar to QuantiFERON&’s for LTBI diagnosis but has better sensitivities among cohorts on immunosuppressive therapies [49].

The urine lipoarabinomannan tuberculosis LAM test was launched in 2015; the test is for active TB diagnosis in HIV‐infected persons with severe immunosuppression (CD4 < 200 cells/μL) [50]. This test was found to possess a sensitivity of between 45% and 60% concerning the activity degree of immunosuppression, while the specificity is about 90%–95% [30, 51, 52]. As stated in the few studies, the test works quickly, typically within 20–30 min, and results can be obtained. However, it is used in immune tolerance states and is ineffective in HIV‐negative or moderately mild immunocompromised states.

Recent studies have identified some antigens associated with latency during MTB dormancy. (DosR‐) encoded antigens are immunogenic. Tests based on measuring antibody responses to these antigens are thought to have the potential to differentiate between latent and active TB [53, 54]. A comparison of TB diagnostic methods is given in Tables 1 and 2.

**TABLE 1:** Comparison of TB diagnostic methods.

Test	QuantiFERON‐TB gold (IGRA)	T‐SPOT.TB (IGRA)	GeneXpert MTB/RIF	Alere determine TB LAM
Type	Immunological	Immunological	NAAT	Immunological
Introduction year	Early 2000s	Early 2000s	2010	2015
Study time	∼24 h	∼24 h	∼2 h	20–30 min
Clinical samples	Blood	Blood	Sputum, CSF (TB meningitis), extrapulmonary samples	Urine
Clinical use	Detects latent TB infection (LTBI)	Detects latent TB infection (LTBI)	Detects TB and rifampicin resistance	Detects active TB in HIV‐positive patients from urine
WHO recommendation	Recommended for LTBI diagnosis in low TB prevalence settings, but not for distinguishing active from latent TB.	Same as QuantiFERON‐TB gold.	Initial diagnostic test in high TB burden areas; also recommended for drug resistance testing.	Recommended for diagnosing TB in severely immunocompromised HIV‐positive patients with CD4 < 200 cells/μL.
Sensitivity & specificity	Sensitivity: 75%–85% (active TB) specificity: 98%–99%	Sensitivity: ∼90% specificity: 98%	Sensitivity: 85%–90% (pulmonary TB) specificity: ∼98%	Sensitivity: 45%–60% (higher in HIV‐positive) specificity: 90%–95%
Advantages	– More specific than TST (tuberculin skin test) – Not affected by BCG vaccination	– Highly sensitive in detecting latent TB – No cross‐reaction with BCG vaccine	– Rapid (results in ∼2 h) – Detects rifampicin resistance – High sensitivity and specificity	– Non‐invasive (urine‐based) – Fast (results in ∼20 min) – Useful in HIV‐positive patients
Disadvantages	– Cannot differentiate between latent and active TB – Requires sophisticated lab infrastructure	– Cannot differentiate between latent and active TB – Lab infrastructure required	– Expensive equipment – Requires stable electricity and infrastructure – Limited in some low‐resource settings	– Low sensitivity in HIV‐negative and non‐severely immunocompromised patients

**TABLE 2:** Comparison of TB diagnostic methods.

Test	TB‐LAMP	GeneXpert ultra	Truenat MTB
Type	NAAT	NAAT	NAAT
Introduction year	2016	2017	2020
Study time	1–2 h	∼2 h	< 1 h
Clinical samples	Sputum	Sputum, CSF, extrapulmonary samples	Sputum
Clinical use	Detects TB from sputum	Detects TB and rifampicin resistance (improved sensitivity)	Detects TB and rifampicin resistance
WHO recommendation	Replacement for smear microscopy in low‐resource settings.	Replacement for GeneXpert MTB/RIF, particularly in HIV‐positive and smear‐negative patients.	Recommended for point‐of‐care use, especially in decentralized and resource‐limited settings.
Sensitivity & specificity	Sensitivity: ∼80% specificity: 98%–99%	Sensitivity: ∼90% (enhanced for HIV‐positive and smear‐negative cases) specificity: ∼98%	Sensitivity: 85%–90% specificity: 98%
Advantages	– Simple and affordable – Suitable for low‐resource settings – No thermal cycler is needed	– Improved sensitivity in smear‐negative and HIV‐positive patients – Same quick turnaround time as GeneXpert MTB/RIF	– Portable and battery‐operated – Suitable for decentralized settings – Quick turnaround (< 1 h)
Disadvantages	– Lower sensitivity compared to GeneXpert – Limited to detecting TB only (no drug resistance detection)	– Same limitations as GeneXpert MTB/RIF regarding cost and infrastructure requirements	– Requires additional testing infrastructure for rifampicin resistance detection

## 5 Nanoparticles Based Point‐of‐Care (POC) Tests

Difficult and laborious methods of TB detection can lead to disease progression. Hence the need for point‐of‐care (POC) tests that provide rapid and sensitive detection. Although several phenotypic colorimetric methods have been actively used for the detection of various microorganisms, efforts on designing nanoparticles (NPs) integrated nano‐biosensors have been performed to prevent the spread of infectious diseases, and early diagnosis through POC testing is essential [55, 56, 57, 58, 59, 60, 61, 62, 63, 64]. The NPs can help prevent the spread of infectious diseases by facilitating the use of biosensors in a variety of environments. The aim is to use NPs in POC tests to develop rapid, sensitive, and cost‐effective detection methods. The development of POC tests with high sensitivity and selectivity facilitated by the large surface area of NPs, enables rapid, portable, and highly stable diagnostic tests [65, 66, 67, 68]. Also, these tests allow the diagnosis to be made on urine, blood, and sputum samples of active TB cases [69]. POC tests can be developed using different mechanisms and NPs, such as simple surface modification of magnetic NPs (MNPs) and generation of resonance signals, and the colorimetric response of gold NPs (Au NPs) with Localized Surface Plasmon Resonance (LSPR) property [65, 70]. In recent years, Au NPs have been actively used in bioanalytical applications and screening tests. Au NPs are preferred in POC assays due to their high stability and durability, simple synthesis methods and structure that allows surface modification [71, 72]. For example, the use of Au NPs in POC‐based colorimetric sensors has been demonstrated in the detection of many pathogens, such as *Staphylococcus aureus* and *Escherichia coli* [73, 74]. On the other hand, rapid analyte determination in POC testing using magnetic field sample separation and reusability features have made the use of magnetic NPs effective [75, 76].

In addition, quantum dots for their high fluorescence brightness, broad color spectrum, and signal enhancement properties; silver NPs (Ag NPs) for their optical and antimicrobial properties are the preferred NPs for POC assays. The NP to be used is selected according to the properties of the target molecule, thus increasing the detection limit and the efficiency of rapid, sensitive and reliable POC tests [77, 78]. NPs‐based colorimetric sensors have shown improvements in MTB detection in terms of high sensitivity and increased specificity. These tests exploit the high surface‐to‐volume ratio of NPs to provide rapid and sensitive detection of the pathogen invisible to the naked eye. Offering an innovative approach to MTB diagnostics, NPs form the basis of colorimetric sensors for the detection of the pathogen [79].

**FIGURE 3:**
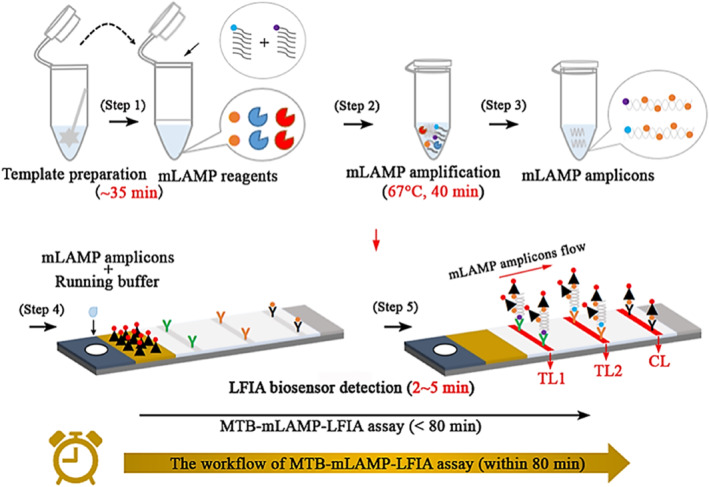
Nanoparticle‐based POC tests for TB diagnostics. Re‐used with permission [79]. Copyright 2023, American Chemical Society.

Yang et al. developed lateral flow MTB assays that can provide rapid and practical results in TB diagnosis. In the test, three primer sets that play an important role in TB diagnosis were designed and synthesized for LAMP, a nucleic acid amplification method used in TB diagnosis. The designed primers were immobilized in LFA test lines, and LFA systems capable of detecting multiple TB biomarkers were developed using multiplex LAMP (mLAMP). The aim was to obtain stronger signals by using polymer NPs in the designed LFA assays. Thus, NP‐based colorimetric POC tests for TB diagnosis at low concentrations are presented. The working principle of the POC test developed for TB detection is shown in Figure 3 [79].

Hussain et al. developed a rational method for the colorimetric assay of TB using unmodified Au NPs. Basically, when there is no target molecule in the environment, ssDNA oligo‐targets cover the surface of Au NPs as a result of electrostatic interaction with the surface of Au NPs and maintain their stability by preventing them from interacting with the salt in the environment. On the other hand, in the presence of target DNA in the environment, the oligo‐target interacts with the target DNA and the unprotected Au NPs are aggregated by interacting with the salt in the environment, then blue color formation is observed as a result of aggregation. These assays, which rely on the push‐pull force based on the surface charge of the Au NPs, enable the colorimetric detection of TB [80].

An example of colorimetric assays using both gold and MNPs in TB diagnostics was developed by Kim et al. For the detection of the TB‐specific antigen CFP‐10, Au NPs and MNPs modified with TB antibodies were used in LFA sensors. The developed POC test enabled effective TB detection at very low concentrations owing to the high surface area/volume ratio feature of NPs. The results obtained were compared with classical methods (PCR and MGIT) and the advantages of the test were demonstrated [14].

Rapid and early diagnosis of TB, which spreads rapidly and has a high mortality rate, is important. Rapid and effective results, ease of use and accessibility provided by POC tests are very important in TB diagnosis. Various NMs (quantum dots, silica NPs, plasmonic NPs, etc.) can be used in the tests to provide colorimetric, sensitive, and rapid detection [81, 82, 83]. The developed TB POC tests have a high commercialization potential.

## 6 Biomarker‐Based Diagnostics

Early and rapid accurate detection of tuberculosis and antibiotic resistance is crucial for the management of tuberculosis, especially in MDR isolates. This can improve outcomes and prevent its spread. In 2014, WHO called for the development of a “rapid biomarker‐based non‐sputum test capable of detecting all forms of TB by identifying characteristic biomarkers” [84]. Several biomarkers can be utilized based on the clinical samples, which are blood, sputum, and urine, for this diagnostic test. Also, active or latent TB can be detected using different biomarkers in this test [85]. Isothermal amplification strategies have the potential to be rapid diagnostic tests due to their accelerated amplification rates (10–60 min) and good applicability [86]. The study reported by Liu et al. on PSR investigation instead of PCR. Another study included a method involving metal ion‐dependent multicomponent nucleic acid enzyme (MNAzyme) developed from DNAzyme [87]. Antigen measurement‐based tests incorporating nanopores and systems incorporating colorimetric sensors with isothermal reactions are currently under development [88].

**FIGURE 4:**
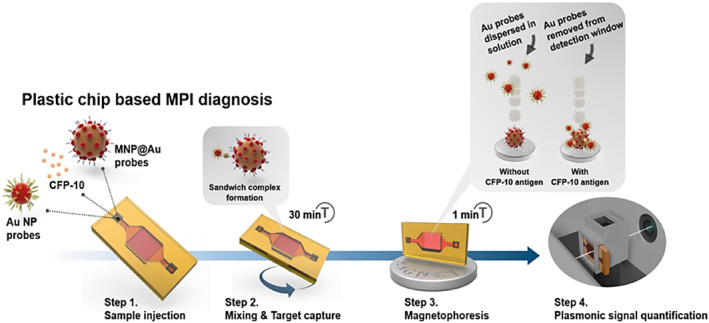
Magnetic‐gold nanoparticles‐based assays for the detection of TB‐specific CFP‐10 antigen tests. Re‐used with permission [14]. Copyright 2016, American Chemical Society.

M. tuberculosis test system based on aptamers is recognized for its high performance in patients with suspected pulmonary tuberculosis [89]. Lavania et al. developed a DNA aptamer‐based immobilized sorbent test and electrochemical sensors and demonstrated high (90%) operability in the diagnosis of active TB. The immobilized sorbent test showed a color change from colorless to blue as a result of oxidation in the presence of TB with 3,3′,5,5′‐tetramethylbenzidine (TMB) added to the plate. This allows easy tracking of the disease with the colorimetric response of the developed test [90]. In another study, Gurmessa et al. targeted antigen‐based tuberculosis detection using a colorimetric enzyme‐linked immunosorbent assay (ELISA) for the detection of CFP‐10, a TB‐specific antigen. The detection limit of CFP‐10 was performed using plasmonic NPs, and the detection limit was obtained from colorimetric responses with TMB oxidation by exploiting the catalytic activity of NPs. The results showed that the developed antigen‐based tests provide a sensitive analytical technique for clinical diagnosis and have a high potential for use in TB diagnosis in the clinic [91]. A rational method using quantum dots and copper NPs (Cu NPs) has been developed to detect LAM, an important biomarker in TB diagnosis. Despite its relatively low sensitivity, LAM is a convenient, non‐invasive method for TB diagnosis. In that study, LAM, a TB biomarker, was detected from urine samples, and the limit of detection studies were performed with high sensitivity thanks to fluorometer readings [24]. Kim et al. developed MNPs‐based assays for the detection of TB‐specific CFP‐10 antigen. The test relies on an antigen‐antibody reaction based on the surface functionalization of magnetic Au NPs [14]. The principle of the developed test is shown in Figure 4 [14]. In addition, Ag85 protein detection is another important biomarker in TB diagnosis. To detect the Ag85B protein, Li et al. used a silicon nanowire field effect transistor (SiNW‐FET) biosensor for *M*. *tuberculosis* in crude sputum [92].

## 7 Diagnostic Methods in Artificial Intelligence Supported by Imaging Techniques

Radiographic images are used to diagnose TB and monitor disease progression. In underdeveloped countries, detection is limited due to lack of resources. However, the characteristics of the devices used in imaging and the lack of an experienced healthcare team create difficulties in accurate detection. Here, artificial intelligence‐assisted diagnosis is proposed to avoid the drawbacks of poor image quality and inexperienced personnel. Diagnosis of TB using radiological images is a highly accurate, sensitive, rapid, and cost‐effective detection method compared with traditional methods. Additionally, it does not require body fluid samples to proccess. There is increasing interest in image processing and artificial intelligence diagnostic methods to overcome diagnostic difficulties [93, 94, 95, 96]^.^

In the study reported by Nijiati et al., chest X‐rays of patients and healthy individuals were analyzed using a deep convolutional neural network (DCNN)‐based artificial intelligence (AI) algorithm to diagnose active pulmonary TB. High accuracy was achieved by using various CNN algorithms, including ResNet, VGG, and AlexNet. According to the results obtained, this easy‐to‐use and low‐cost diagnostic method has the potential for widespread use [97]. Another study on AI‐assisted TB diagnosis was conducted by Jamal et al. In that study, the diagnosis of resistant and susceptible mutations was performed using artificial intelligence and machine learning techniques. The sequences in the rpoB, inhA, katG, pncA, gyrA, and gyrB genes of several drugs were used as a data set. By analyzing the mutation occurring in the genes with artificial intelligence‐supported systems, diagnostic methods that are important for the treatment of the disease have been developed [98].

## 8 Future Perspectives

The molecular methods still have advantages in the diagnosis and management of TB disease, including high throughput, short response time, and accurate results compared to conventional methods. In contrast to that, these methods have some limitations, such as complicated procedures, expensiveness, need for trained staff, and partial lack of sensitivity of detection of drug resistance (except GeneXpert MTB/RIF or GeneXpert Ultra method). POC tests that can be used by patients themselves are expected to become widespread, as well as tests that are rapid, sensitive, highly accurate, and capable of colorimetric response. In these tests, the use of NPs, which increase the sensitivity of the tests, is receiving attention in diagnosis and management of active TB patients. The NPs incorporated tests may offer the advantage of indirect detection of TB biomarkers and may increase the potential for future use of rapid TB tests.

## 9 Conclusion

Various TB diagnostic tests, such as NAATs, immunological tests, and culture‐alternative methods, are expected to have higher sensitivity, specificity, and speed than traditional laboratory methods of TB diagnosis, such as culture and smear microscopy. GeneXpert Ultra and Truenat have been universally accepted tools for TB diagnosis, including drug resistance approved by WHO. On the other hand, IGRAs are still efficient in the diagnosis of latent TB in a situation of low disease burden. The development of cost‐effective, simple, rapid, and sensitive tests with easy‐to‐use POC tests shows great promise for TB diagnosis. The use of image‐based diagnosis with developing technology is very important for treatment. All newly developed TB tests offer potential convenience for the early detection and control of TB worldwide.

## Author Contributions

Nimet Temur, Esma Eryilmaz‐ Eren, Ilhami Celik, Ilknur E. Yıldız, Mustafa Nisari, Ilay S. Unal, Cagla Celik, Nilay Ildiz and Ismail Ocsoy wrote the manuscript. Nimet Temur prepared the figures. Ismail Ocsoy supervised the manuscript.

## Conflicts of Interest

The authors declare no conflicts of interest.

## Data Availability

The data supporting the results of this study are available on request from the corresponding author. The data are not publicly available due to privacy or ethical restrictions.

## References

[1] Global Tuberculosis Report 2023 (World Health Organization, 2023).

[2] Methods Used By WHO To Estimate The Global Burden of TB Disease (World Health Organization, 2021).

[3] X. Zhang , Y. Tian , Y. Shi , et al., “Naked‐Eye LAMP Assay of M. tuberculosis in Sputum by In Situ Au Nanoprobe Identification: For the In Vitro Diagnostics of Tuberculosis,” ACS Infectious Diseases 10 (2024): 2668–2678. 38771809 10.1021/acsinfecdis.4c00013

[4] R. Brosch , S. V. Gordon , M. Marmiesse , et al., “A New Evolutionary Scenario for the *Mycobacterium tuberculosis* Complex,” Proceedings of the National Academy of Sciences 99 (2002): 3684–3689. 10.1073/pnas.052548299 PMC122584 11891304

[5] S. T. Cole , R. Brosch , J. Parkhill , et al., “Deciphering the Biology of *Mycobacterium tuberculosis* From the Complete Genome Sequence,” Nature 393 (1998): 537–544. 9634230 10.1038/31159

[6] M. M. Bigi , M. A. Forrellad , J. S. García , F. C. Blanco , C. L. Vázquez , and F. Bigi , “An Update on *Mycobacterium tuberculosis* Lipoproteins,” Future Microbiology 18 (2023): 1381–1398. 37962486 10.2217/fmb-2023-0088

[7] B. P. Kelly , S. K. Furney , M. T. Jessen , and I. M. Orme , “Low‐Dose Aerosol Infection Model for Testing Drugs for Efficacy Against *Mycobacterium tuberculosis* ,” Antimicrobial Agents and Chemotherapy 40 (1996): 2809–2812. 9124846 10.1128/aac.40.12.2809 PMC163627

[8] A. N. Leung , “Pulmonary Tuberculosis: The Essentials,” Radiology 210 (1999): 307–322. 10207408 10.1148/radiology.210.2.r99ja34307

[9] I. Smith , “ *Mycobacterium tuberculosis* Pathogenesis and Molecular Determinants of Virulence,” Clinical Microbiology Reviews 16 (2003): 463–496. 12857778 10.1128/CMR.16.3.463-496.2003 PMC164219

[10] R. D. Turner and G. H. Bothamley , “Cough and the Transmission of Tuberculosis,” Journal of Infectious Diseases 211 (2015): 1367–1372. 25387581 10.1093/infdis/jiu625

[11] S. D. Shi , P. R. Hsueh , P. C. Yang , and C. C. Chou , “Use of DosR Dormancy Antigens From *Mycobacterium tuberculosis* for Serodiagnosis of Active and Latent Tuberculosis,” ACS Infectious Diseases 6 (2019): 272–280. 31815418 10.1021/acsinfecdis.9b00329

[12] U. Tansuphaisiri and B. Kladphuang , “Evaluation of Sputum Staining by Modified Cold Method and Comparison With Ziehl‐Neelsen and Fluorochrome Methods for the Primary Diognosis of Tuberculosis,” Southeast Asian Journal of Tropical Medicine and Public Health 33 (2002): 128–135. 12118440

[13] C. Abe, “Standardization of Laboratory Tests for Tuberculosis and Their Proficiency Testing,” Kekkaku 78 (2003): 541–551. 14509226

[14] J. Kim , M. Jang , K. G. Lee , et al., “Plastic‐Chip‐Based Magnetophoretic Immunoassay for Point‐of‐Care Diagnosis of Tuberculosis,” ACS Applied Materials & Interfaces 8 (2016): 23489–23497. 27548010 10.1021/acsami.6b06924

[15] Y. J. Ryu , “Diagnosis of Pulmonary Tuberculosis: Recent Advances and Diagnostic Algorithms,” Tuberculosis and Respiratory Diseases 78 (2015): 64–71. 25861338 10.4046/trd.2015.78.2.64 PMC4388902

[16] R. Loddenkemper , M. Lipman , and A. Zumla , “Clinical Aspects of Adult Tuberculosis,” Cold Spring Harbor Perspectives in Medicine 6 (2016): a017848. 10.1101/cshperspect.a017848 PMC4691808 25659379

[17] L. Muñoz , H. R. Stagg , and I. Abubakar , “Diagnosis and Management of Latent Tuberculosis Infection: Table 1,” Cold Spring Harbor Perspectives in Medicine 5 (2015): a017830. 26054858 10.1101/cshperspect.a017830 PMC4632867

[18] D. Brodie and N. W. Schluger , “The Diagnosis of Tuberculosis,” Clinics in Chest Medicine 26 (2005): 247–271. 15837109 10.1016/j.ccm.2005.02.012

[19] T. A. Campelo , P. R. Cardoso de Sousa , L. L. Nogueira , C. C. Frota , and P. R. Zuquim Antas , “Revisiting the Methods for Detecting *Mycobacterium tuberculosis*: What Has the New Millennium Brought Thus Far?,” Access Microbiology 3 (2021): 000245. 34595396 10.1099/acmi.0.000245 PMC8479963

[20] D. A. Moore , C. A. Evans , R. H. Gilman , et al., “Microscopic‐Observation Drug‐Susceptibility Assay for the Diagnosis of TB,” New England Journal of Medicine 355 (2006): 1539–1550. 17035648 10.1056/NEJMoa055524 PMC1780278

[21] N. S. Shah , P. Moodley , P. Babaria , et al., “Rapid Diagnosis of Tuberculosis and Multidrug Resistance by the Microscopic‐Observation Drug‐Susceptibility Assay,” American Journal of Respiratory and Critical Care Medicine 183 (2011): 1427–1433. 21297071 10.1164/rccm.201009-1449OC

[22] Y. Boum , P. Orikiriza , G. Rojas‐Ponce , et al., “Use of Colorimetric Culture Methods for Detection of *Mycobacterium tuberculosis* Complex Isolates From Sputum Samples in Resource‐Limited Settings,” Journal of Clinical Microbiology 51 (2013): 2273–2279. 23658270 10.1128/JCM.00749-13 PMC3697731

[23] A. Gupta , M. R. Sen , T. M. Mohapatra , and S. Anupurba , “Evaluation of the Performance of Nitrate Reductase Assay for Rapid Drug‐Susceptibility Testing of *Mycobacterium tuberculosis* in North India,” Journal of Health, Population, and Nutrition 29 (2011): 20–25. 21528787 10.3329/jhpn.v29i1.7563 PMC3075052

[24] P. Chen , Y. Meng , T. Liu , et al., “Sensitive Urine Immunoassay for Visualization of Lipoarabinomannan for Noninvasive Tuberculosis Diagnosis,” ACS Nano 17 (2023): 6998–7006. 37010068 10.1021/acsnano.3c01374

[25] P. Chen , M. Shi , G. D. Feng , et al., “A Highly Efficient Ziehl‐Neelsen Stain: Identifying *De Novo* Intracellular *Mycobacterium tuberculosis* and Improving Detection of Extracellular *M. tuberculosis* in Cerebrospinal Fluid,” Journal of Clinical Microbiology 50 (2012): 1166–1170. 22238448 10.1128/JCM.05756-11 PMC3318527

[26] G. Theron , R. Venter , G. Calligaro , et al., “Xpert MTB/RIF Results in Patients With Previous Tuberculosis: Can We Distinguish True From False Positive Results?,” Clinical Infectious Diseases 62 (2016): 995–1001. 26908793 10.1093/cid/civ1223 PMC4803105

[27] Y. R. Ngangue , C. Mbuli , A. Neh , et al., “Diagnostic Accuracy of the Truenat MTB Plus Assay and Comparison With the Xpert MTB/RIF Assay to Detect Tuberculosis Among Hospital Outpatients in Cameroon,” Journal of Clinical Microbiology 60 (2022): e00155‐22. 35861529 10.1128/jcm.00155-22 PMC9383115

[28] O. P. R. Balisan , J. R. T. Galamay , L. N. Cale‐Subia , and A. M. De Luna , “Utility of Nucleic Acid Amplification Test in the Detection of Tuberculosis in Biological Fluids From Suspected TB Patients in a Cardiovascular Center in the Philippines,” Acta Tropica 249 (2024): 107078. 38036022 10.1016/j.actatropica.2023.107078

[29] L. R. Inbaraj , J. Daniel , M. K. Sathya Narayanan , et al., “TB‐LAMP (Loop‐Mediated Isothermal Amplification) for Diagnosing Pulmonary Tuberculosis in Children,” Cochrane Database of Systematic Reviews 2023 (2023): CD015806. 10.1002/14651858.CD015806.pub2 PMC12188635 40557818

[30] N. Zaporojan , R. A. Negrean , R. Hodișan , C. Zaporojan , A. Csep , and D. C. Zaha , “Evolution of Laboratory Diagnosis of Tuberculosis,” Clinics and Practice 14 (2024): 388–416. 38525709 10.3390/clinpract14020030 PMC10961697

[31] R. P. Mahato and S. Kumar , “The Future in Diagnostic Tools for TB Outbreaks: A Review of the Approaches With Focus on LAMP and RPA Diagnostics Tests,” Journal of Microbiological Methods 227 (2024): 107064. 39448035 10.1016/j.mimet.2024.107064

[32] L. R. Inbaraj , J. Daniel , P. Rajendran , et al., “Truenat MTB Assays for Pulmonary Tuberculosis and Rifampicin Resistance in Adults,” Cochrane Database of Systematic Reviews 2023 (2023): CD015543. 10.1002/14651858.CD015543.pub2 PMC11930391 40122135

[33] Y. C. Yang , P. L. Lu , S. C. Huang , Y. S. Jenh , R. Jou , and T. C. Chang , “Evaluation of the Cobas TaqMan MTB Test for Direct Detection of *Mycobacterium tuberculosis* Complex in Respiratory Specimens,” Journal of Clinical Microbiology 49 (2011): 797–801. 21177901 10.1128/JCM.01839-10 PMC3067742

[34] R. L. Guerra , N. M. Hooper , J. F. Baker , et al., “Use of the Amplified *Mycobacterium tuberculosis* Direct Test in a Public Health Laboratory,” Chest 132 (2007): 946–951. 17573496 10.1378/chest.06-2959

[35] M. Pai , S. Kalantri , and K. Dheda , “New Tools and Emerging Technologies for the Diagnosis of Tuberculosis: Part II. Active Tuberculosis and Drug Resistance,” Expert Review of Molecular Diagnostics 6 (2006): 423–432. 16706744 10.1586/14737159.6.3.423

[36] T. Notomi , Y. Mori , N. Tomita , and H. Kanda , “Loop‐mediated Isothermal Amplification (LAMP): Principle, Features, and Future Prospects,” Journal of Microbiology 53 (2015): 1–5. 25557475 10.1007/s12275-015-4656-9

[37] Y. P. Wong , S. Othman , Y. L. Lau , S. Radu , and H. Y. Chee , “Loop‐Mediated Isothermal Amplification (LAMP): A Versatile Technique for Detection of Micro‐organisms,” Journal of Applied Microbiology 124 (2018): 626–643. 29165905 10.1111/jam.13647 PMC7167136

[38] “Nationwide Shortage of Tuberculin Skin Test Antigens: CDC Recommendations for Patient Care and Public Health Practice,” Morbidity and Mortality Weekly Report 68 (2019): 552–553. 31220054 10.15585/mmwr.mm6824a4 PMC6586373

[39] R. A. Pennie , “Mantoux Tests. Performing, Interpreting, and Acting Upon Them,” Canadian Family Physician 41 (1995): 1025–1029. 7780314 PMC2146560

[40] A. A. Lardizabal and L. B. Reichman , “Diagnosis of Latent Tuberculosis Infection,” Microbiology Spectrum 5 (2017): TNMI7-0019-2016. 10.1128/microbiolspec.tnmi7-0019-2016 PMC11687433 28185619

[41] R. E. Huebner , M. F. Schein , and J. B. Bass Jr., “The Tuberculin Skin Test,” Clinical Infectious Diseases 17 (1993): 968–975. 8110954 10.1093/clinids/17.6.968

[42] P. L. Lin , S. Pawar , A. Myers , et al., “Early Events in *Mycobacterium tuberculosis* Infection in Cynomolgus Macaques,” Infection and Immunity 74 (2006): 3790–3803. 16790751 10.1128/IAI.00064-06 PMC1489679

[43] W. Bae , K. U. Park , E. Y. Song , et al., “Comparison of the Sensitivity of QuantiFERON‐TB Gold In‐Tube and T‐SPOT.TB According to Patient Age,” PLoS ONE 11 (2016): e0156917. 27258377 10.1371/journal.pone.0156917 PMC4892501

[44] G. H. Mazurek , J. Jereb , P. LoBue , M. F. Iademarco , B. Metchock , and A. Vernon , “Guidelines for Using the QuantiFERON®‐TB Gold Test for Detecting *Mycobacterium tuberculosis* Infection, United States,” Morbidity and Mortality Weekly Report Recommendations and Reports 54 (2005): 49–55. 16357824

[45] T. Kurenuma , I. Kawamura , H. Hara , et al., “The RD1 Locus in the *Mycobacterium tuberculosis* Genome Contributes to Activation of Caspase‐1 via Induction of Potassium Ion Efflux in Infected Macrophages,” Infection and Immunity 77 (2009): 3992–4001. 19596775 10.1128/IAI.00015-09 PMC2737993

[46] S. Daugelat , J. Kowall , J. Mattow , et al., “The RD1 Proteins of *Mycobacterium tuberculosis*: Expression in *Mycobacterium smegmatis* and Biochemical Characterization,” Microbes and Infection 5 (2003): 1082–1095. 14554249 10.1016/s1286-4579(03)00205-3

[47] A. Shafeque , J. Bigio , C. A. Hogan , M. Pai , and N. Banaei , “Fourth‐Generation QuantiFERON‐TB Gold Plus: What Is the Evidence?,” Journal of Clinical Microbiology 58 (2020): e01950‐19. 32493779 10.1128/JCM.01950-19 PMC7448650

[48] Y. Ma , R. Li , J. Shen , et al., “Clinical Effect of T‐SPOT.TB Test for the Diagnosis of Tuberculosis,” BMC Infectious Diseases 19 (2019): 993. 31752713 10.1186/s12879-019-4597-8 PMC6873440

[49] The Use of Lateral Flow Urine Lipoarabinomannan Assay (LF‐LAM) for the Diagnosis and Screening of Active Tuberculosis in People Living With HIV: Policy Guidance (World Health Organization, 2015).

[50] J. Peter , G. Theron , D. Chanda , et al., “Test Characteristics and Potential Impact of the Urine LAM Lateral Flow Assay in HIV‐Infected Outpatients Under Investigation for TB and Able to Self‐Expectorate Sputum for Diagnostic Testing,” BMC Infectious Diseases 15 (2015): 262. 26156025 10.1186/s12879-015-0967-z PMC4495934

[51] A. Benjamin , S. C. Cavalcante , L. F. Jamal , et al., “Accuracy of Determine TB‐LAM Ag to Detect TB in HIV Infected Patients Associated With Diagnostic Methods Used in Brazilian Public Health Units,” PLoS One 14 (2019): e0221038. 31550246 10.1371/journal.pone.0221038 PMC6759169

[52] S. Singh , I. Saraav , and S. Sharma , “Immunogenic Potential of Latency Associated Antigens Against *Mycobacterium tuberculosis* ,” Vaccine 32 (2014): 712–716. 24300592 10.1016/j.vaccine.2013.11.065

[53] I. Latorre and J. Domínguez , “Dormancy Antigens as Biomarkers of Latent Tuberculosis Infection,” EBioMedicine 2 (2015): 790–791. 26425678 10.1016/j.ebiom.2015.06.017 PMC4563125

[54] K. Dheda , M. Ruhwald , G. Theron , J. Peter , and W. C. Yam , “Point‐Of‐Care Diagnosis of Tuberculosis: Past, Present and Future,” Respirology 18 (2013): 217–232. 23190246 10.1111/resp.12022

[55] A. L. García‐Basteiro , A. Dinardo , B. Saavedra , et al., “Point of Care Diagnostics for Tuberculosis,” Pulmonology 24 (2018): 73–85. 29426581 10.1016/j.rppnen.2017.12.002

[56] J. M. Hong , H. Lee , N. V. Menon , C. T. Lim , L. P. Lee , and C. W. Ong , “Point‐Of‐Care Diagnostic Tests for Tuberculosis Disease,” Science Translational Medicine 14 (2022): eabj4124. 35385338 10.1126/scitranslmed.abj4124

[57] A. K. Gupta , A. Singh , and S. Singh , “Diagnosis of Tuberculosis: Nanodiagnostics Approaches,” in NanoBioMedicine, ed. S. K. Saxena and S. M. P. Khurana (Springer, 2020), 261–283.

[58] C. Celik , N. Ildiz , M. Z. Kaya , A. B. Kilic , and I. Ocsoy , “Preparation of Natural Indicator Incorporated Media and its Logical Use as a Colorimetric Biosensor for Rapid and Sensitive Detection of Methicillin‐Resistant Staphylococcus aureus,” Analytica Chimica Acta 1128 (2020): 80–89. 32825915 10.1016/j.aca.2020.06.005

[59] C. Celik , N. Ildiz , P. Sagiroglu , M. A. Atalay , C. Yazici , and I. Ocsoy , “Preparation of Nature Inspired Indicator Based Agar for Detection and Identification of MRSA and MRSE,” Talanta 219 (2020): 121292. 32887034 10.1016/j.talanta.2020.121292

[60] C. Celik , N. Y. Demir , M. Duman , N. Ildiz , and I. Ocsoy , “Red Cabbage Extract‐Mediated Colorimetric Sensor for Swift, Sensitive and Economic Detection of Urease‐Positive Bacteria by Naked Eye and Smartphone Platform,” Scientific Reports 13 (2023): 2056. 36739311 10.1038/s41598-023-28604-1 PMC9899230

[61] C. Celik , G. Can Sezgin , U. G. Kocabas , et al., “Novel Anthocyanin‐Based Colorimetric Assay for the Rapid, Sensitive, and Quantitative Detection of *Helicobacter pylori* ,” Analytical Chemistry 93 (2021): 6246–6253. 33825433 10.1021/acs.analchem.1c00663

[62] C. Celik , G. Kalin , Z. Cetinkaya , N. Ildiz , and I. Ocsoy , “Recent Advances in Colorimetric Tests for the Detection of Infectious Diseases and Antimicrobial Resistance,” Diagnostics 13 (2023): 2427. 37510171 10.3390/diagnostics13142427 PMC10377832

[63] G. Can Sezgin , Y. E. Tekin , B. Calim , et al., “Development of a Colorimetric Urease Test Based on Au NPS Capped With Anthocyanin for the Rapid Detection of *Helicobacter pylori* Through Multiple Readouts,” ChemistrySelect 8 (2023): e202300637.

[64] M. I. Shukoor , M. O. Altman , D. Han , et al., “Aptamer‐Nanoparticle Assembly for Logic‐Based Detection,” ACS Applied Materials & Interfaces 4 (2012): 3007–3011. 22650355 10.1021/am300374q PMC3483412

[65] T. T. Tsai , C. Y. Huang , C. A. Chen , et al., “Diagnosis of Tuberculosis Using Colorimetric Gold Nanoparticles on a Paper‐Based Analytical Device,” ACS Sensors 2 (2017): 1345–1354. 28901134 10.1021/acssensors.7b00450

[66] S. Nandini , S. Nalini , S. Bindhu , et al., “Current Trends of Functionalized Nanomaterial‐Based Sensors in Point‐Of‐Care Diagnosis,” in Functionalized Nanomaterial‐Based Electrochemical Sensors, ed. C. M. Hussain and J. G. Manjunatha (Woodhead Publishing, 2022), 337–353

[67] J. Ma , M. Du , C. Wang , et al., “Rapid and Sensitive Detection of *Mycobacterium tuberculosis* by an Enhanced Nanobiosensor,” ACS Sensors 6 (2021): 3367–3376. 34470206 10.1021/acssensors.1c01227

[68] J. Kim , V. T. Tran , S. Oh , et al., “Clinical Trial: Magnetoplasmonic ELISA for Urine‐Based Active Tuberculosis Detection and Anti‐tuberculosis Therapy Monitoring,” ACS Central Science 7 (2021): 1898–1907. 34841060 10.1021/acscentsci.1c00948 PMC8614099

[69] Y. Wang , L. Yu , X. Kong , and L. Sun , “Application of Nanodiagnostics in Point‐Of‐Care Tests for Infectious Diseases,” International Journal of Nanomedicine 12 (2017): 4789–4803. 28740385 10.2147/IJN.S137338 PMC5503494

[70] M. Cordeiro , F. Ferreira Carlos , P. Pedrosa , A. Lopez , and P. V. Baptista , “Gold Nanoparticles for Diagnostics: Advances Towards Points of Care,” Diagnostics 6 (2016): 43. 27879660 10.3390/diagnostics6040043 PMC5192518

[71] Y. Gupta and A. S. Ghrera , “Recent Advances in Gold Nanoparticle‐Based Lateral Flow Immunoassay for the Detection of Bacterial Infection,” Archives of Microbiology 203 (2021): 3767–3784. 34086107 10.1007/s00203-021-02357-9

[72] X. He , T. Hao , H. Geng , et al., “Sensitization Strategies of Lateral Flow Immunochromatography for Gold Modified Nanomaterials in Biosensor Development,” International Journal of Nanomedicine 18 (2023): 7847–7863. 38146466 10.2147/IJN.S436379 PMC10749510

[73] X. Z. Mou , X. Y. Chen , J. Wang , et al., “Bacteria‐Instructed Click Chemistry Between Functionalized Gold Nanoparticles for Point‐of‐Care Microbial Detection,” ACS Applied Materials & Interfaces 11 (2019): 23093–23101. 31184853 10.1021/acsami.9b09279

[74] D. Cam and H. A. Öktem , “Optimizations Needed for Lateral Flow Assay for Rapid Detection of Pathogenic E. Coli,” Turkish Journal of Biology 41 (2017): 954–968. 30814860 10.3906/biy-1705-50 PMC6353270

[75] Y. Xianyu , Q. Wang , and Y. Chen , “Magnetic Particles‐Enabled Biosensors for Point‐Of‐Care Testing,” TrAC Trends in Analytical Chemistry 106 (2018): 213–224.

[76] S. Sahoo , A. Nayak , A. Gadnayak , et al., “Quantum Dots Enabled Point-of-Care Diagnostics: A New Dimension to the Nanodiagnosis,” in Advanced Nanomaterials for Point of Care Diagnosis and Therapy, ed. S. Dave , J. Das , and S. Ghosh (Elseiver, 2022), 43–52

[77] F. Beck , M. Loessl , and A. J. Baeumner , “Signaling Strategies of Silver Nanoparticles in Optical and Electrochemical Biosensors: Considering Their Potential for the Point‐Of‐Care,” Microchimica Acta 190 (2023): 91. 36790481 10.1007/s00604-023-05666-6 PMC9930094

[78] S. Roy , F. Arshad , S. Eissa , et al., “Recent Developments Towards Portable Point‐of‐Care Diagnostic Devices for Pathogen Detection,” Sensors & Diagnostics 1 (2022): 87–105.

[79] X. Yang , X. Chen , J. Huang , et al., “Ultrafast, One‐step, Label‐Based Biosensor Diagnosis Platform for the Detection of *Mycobacterium tuberculosis* in Clinical Applications,” ACS Infectious Diseases 9 (2023): 762–772. 36926845 10.1021/acsinfecdis.2c00475

[80] M. M. Hussain , T. M. Samir , and H. M. Azzazy , “Unmodified Gold Nanoparticles for Direct and Rapid Detection of *Mycobacterium tuberculosis* Complex,” Clinical Biochemistry 46 (2013): 633–637. 23318577 10.1016/j.clinbiochem.2012.12.020

[81] N. Patnaik and R. J. Dey , “Label‐Free Citrate‐Stabilized Silver Nanoparticles‐Based, Highly Sensitive, Cost‐Effective, and Rapid Visual Method for the Differential Detection of *Mycobacterium tuberculosis* and *Mycobacterium Bovis* ,” ACS Infectious Diseases 10 (2023): 426–435. 38112513 10.1021/acsinfecdis.3c00390

[82] C. Sun , X. Zhang , J. Wang , Y. Chen , and C. Meng , “Novel Mesoporous Silica Nanocarriers Containing Gold; a Rapid Diagnostic Tool for Tuberculosis,” BMC Complementary Medicine and Therapies 21 (2021): 277. 34740364 10.1186/s12906-021-03451-7 PMC8569953

[83] B. Zhou , M. Zhu , Y. Hao , and P. Yang , “Potential‐Resolved Electrochemiluminescence for Simultaneous Determination of Triple Latent Tuberculosis Infection Markers,” ACS Applied Materials & Interfaces 9 (2017): 30536–30542. 28828860 10.1021/acsami.7b10343

[84] High‐Priority Target Product Profiles for New Tuberculosis Diagnostics: Report of A Consensus Meeting (World Health Organization, 2014).

[85] E. Kamra , T. Prasad , A. Rais , et al., “Diagnosis of Genitourinary Tuberculosis: Detection of Mycobacterial Lipoarabinomannan and MPT‐64 Biomarkers Within Urine Extracellular Vesicles by Nano‐Based Immuno‐PCR Assay,” Scientific Reports 13 (2023): 11560. 37463964 10.1038/s41598-023-38740-3 PMC10354090

[86] B. B. Oliveira , B. Veigas , and P. V. Baptista , “Isothermal Amplification of Nucleic Acids: The Race for the Next ‘Gold Standard’,” Frontiers in Sensors 2 (2021): 752600.

[87] W. Liu , D. Zou , X. He , et al., “Development and Application of a Rapid *Mycobacterium tuberculosis* Detection Technique Using Polymerase Spiral Reaction,” Scientific Reports 8 (2018): 3003. 29445235 10.1038/s41598-018-21376-z PMC5813102

[88] O. Hu , Z. Li , J. Wu , Y. Tan , Z. Chen , and Y. Tong , “A Multicomponent Nucleic Acid Enzyme‐Cleavable Quantum Dot Nanobeacon for Highly Sensitive Diagnosis of Tuberculosis With the Naked Eye,” ACS Sensors 8 (2023): 254–262. 36579361 10.1021/acssensors.2c02114

[89] D. R. Martin , N. R. Sibuyi , P. Dube , et al., “Aptamer‐Based Diagnostic Systems for the Rapid Screening of TB at the Point‐of‐Care,” Diagnostics 11 (2021): 1352. 34441287 10.3390/diagnostics11081352 PMC8391981

[90] S. Lavania , R. Das , A. Dhiman , et al., “Aptamer‐Based TB Antigen Tests for the Rapid Diagnosis of Pulmonary Tuberculosis: Potential Utility in Screening for Tuberculosis,” ACS Infectious Diseases 4 (2018): 1718–1726. 30350564 10.1021/acsinfecdis.8b00201

[91] S. K. Gurmessa , L. T. Tufa , J. Kim , et al., “Colorimetric Detection of *Mycobacterium tuberculosis* ESX‐1 Substrate Protein in Clinical Samples Using Au@Pd Nanoparticle‐Based Magnetic Enzyme‐Linked Immunosorbent Assay,” ACS Applied Nano Materials 4 (2020): 539–549.

[92] H. Li , D. Li , H. Chen , et al., “Application of Silicon Nanowire Field Effect Transistor (SiNW‐FET) Biosensor With High Sensitivity,” Sensors 23 (2023): 6808. 37571591 10.3390/s23156808 PMC10422280

[93] W. Y. N. Naing and Z. Z. Htike , “Advances in Automatic Tuberculosis Detection in Chest X‐Ray Images,” Signal & Image Processing 5 (2014): 41.

[94] R. Hooda , S. Sofat , S. Kaur , A. Mittal , and F. Meriaudeau , “Deep‐Learning: A Potential Method for Tuberculosis Detection Using Chest Radiography,” in IEEE International Conference on Signal and Image Processing Applications (ICSIPA), (IEEE, 2017), 497–502.

[95] P. Lakhani and B. Sundaram , “Deep Learning at Chest Radiography: Automated Classification of Pulmonary Tuberculosis by Using Convolutional Neural Networks,” Radiology 284 (2017): 574–582. 28436741 10.1148/radiol.2017162326

[96] Y. Zhan , Y. Wang , W. Zhang , B. Ying , and C. Wang , “Diagnostic Accuracy of the Artificial Intelligence Methods in Medical Imaging for Pulmonary Tuberculosis: A Systematic Review and Meta‐Analysis,” Journal of Clinical Medicine 12 (2022): 303. 36615102 10.3390/jcm12010303 PMC9820940

[97] M. Nijiati , J. Ma , C. Hu , et al., “Artificial Intelligence Assisting the Early Detection of Active Pulmonary Tuberculosis From Chest X‐Rays: A Population‐Based Study,” Frontiers in Molecular Biosciences 9 (2022): 874475. 35463963 10.3389/fmolb.2022.874475 PMC9023793

[98] S. Jamal , M. Khubaib , R. Gangwar , S. Grover , A. Grover , and S. E. Hasnain , “Artificial Intelligence and Machine Learning Based Prediction of Resistant and Susceptible Mutations in *Mycobacterium tuberculosis* ,” Scientific Reports 10 (2020): 5487. 32218465 10.1038/s41598-020-62368-2 PMC7099008

